# Network topology-based detection of differential gene regulation and regulatory switches in cell metabolism and signaling

**DOI:** 10.1186/1752-0509-8-56

**Published:** 2014-05-16

**Authors:** Rosario M Piro, Stefan Wiesberg, Gunnar Schramm, Nico Rebel, Marcus Oswald, Roland Eils, Gerhard Reinelt, Rainer König

**Affiliations:** 1 Division of Theoretical Bioinformatics, German Cancer Research Center (Deutsches Krebsforschungszentrum, DKFZ), Heidelberg, Germany; 2 Department of Bioinformatics and Functional Genomics, Institute of Pharmacy and Molecular Biotechnology, BioQuant, University of Heidelberg, Heidelberg, Germany; 3 Institute of Computer Science and Interdisciplinary Center for Scientific Computing, University of Heidelberg, Heidelberg, Germany; 4 Center for Sepsis Control and Care, University Hospital Jena, Jena, Germany; 5 Hans-Knöll-Institute (HKI), Jena, Germany; 6 Present address: German Consortium for Translational Cancer Research (DKTK) and Division of Molecular Genetics, German Cancer Research Center (Deutsches Krebsforschungszentrum, DKFZ), Heidelberg, Germany

**Keywords:** Pathway analysis, Network topology, Pathway networks, Gene expression

## Abstract

**Background:**

Common approaches to pathway analysis treat pathways merely as lists of genes disregarding their topological structures, that is, ignoring the genes' interactions on which a pathway's cellular function depends. In contrast, PathWave has been developed for the analysis of high-throughput gene expression data that explicitly takes the topology of networks into account to identify both global dysregulation of and localized (switch-like) regulatory shifts within metabolic and signaling pathways. For this purpose, it applies adjusted wavelet transforms on optimized 2D grid representations of curated pathway maps.

**Results:**

Here, we present the new version of PathWave with several substantial improvements including a new method for optimally mapping pathway networks unto compact 2D lattice grids, a more flexible and user-friendly interface, and pre-arranged 2D grid representations. These pathway representations are assembled for several species now comprising *H. sapiens*, *M. musculus*, *D. melanogaster*, *D. rerio*, *C. elegans*, and *E. coli*. We show that PathWave is more sensitive than common approaches and apply it to RNA-seq expression data, identifying crucial metabolic pathways in lung adenocarcinoma, as well as microarray expression data, identifying pathways involved in longevity of *Drosophila*.

**Conclusions:**

PathWave is a generic method for pathway analysis complementing established tools like GSEA, and the update comprises efficient new features. In contrast to the tested commonly applied approaches which do not take network topology into account, PathWave enables identifying pathways that are either known be involved in or very likely associated with such diverse conditions as human lung cancer or aging of *D. melanogaster*. The PathWave R package is freely available at http://www.ichip.de/software/pathwave.html.

## Background

Large-scale gene expression profiling by microarrays or transcript sequencing enables identifying relevant expression changes in cells by comparing gene expression patterns of two distinct conditions (e.g. tumor versus normal tissue). A frequent goal of such studies is the identification of dysregulated cellular pathways involved in an observed phenotype like, for example, abnormal proliferation and migration of cancer cells.

For this, gene set enrichment tests are commonly applied on large scale gene expression profiles, testing over-representation of up- or/and downregulated genes in pathways. While most gene set enrichment approaches used for pathway analysis ignore topological information, we have introduced a computational method (PathWave) [1] that explicitly takes the topology of metabolic and signaling networks into account by applying adjusted wavelet transforms on optimized, compact 2D grid representations of curated pathway maps, like those from the Kyoto Encyclopedia of Genes and Genomes (KEGG) [2]. In particular, this approach allows identifying not only metabolic pathways that show differential regulation as a whole, but also pathways affected by specific, localized switch-like regulatory shifts indicating a redirection of metabolic fluxes. This way, we have identified important metabolic switches in neuroblastoma [1], breast cancer [3], Alzheimer's disease [4], glioblastoma [5] and in an evolutionary study of *E. coli*[6].

Here, we present the new, entirely revised and refined version of PathWave with several substantial improvements:

i. a faster and more efficient arrangement of 2D grid representations of pathway networks (see below),

ii. pre-arranged 2D grid representations for several species, including *H. sapiens*, *M. musculus*, *D. melanogaster*, *D. rerio*, *C. elegans*, and *E. coli*,

iii. compliance with version 0.7.1 of the KEGG Markup Language (KGML),

iv. an interface to obtain colored KEGG pathway maps depicting the identified expression changes,

v. the use of pathways extracted from the Human metabolic model reconstruction [7] from the Biochemical Genetic and Genomic (BiGG) knowledgebase [8],

vi. performance improvements due to faster processing of expression data, and

vii. a more flexible and user-friendly interface.

We briefly summarize the PathWave workflow, discuss the most important novelties of the new version and demonstrate its usage by two case studies: (i) we applied PathWave to RNA-seq expression data to identify metabolic pathways that play a role in lung cancer, and (ii) we analyzed microarray expression data to identify pathways associated with longevity of *Drosophila*. Finally, we show that PathWave is more sensitive than the frequently used methods Gene Set Enrichment Analysis (GSEA) [9] and DAVID [10].

## Implementation

### PathWave workflow

**Figure 1:**
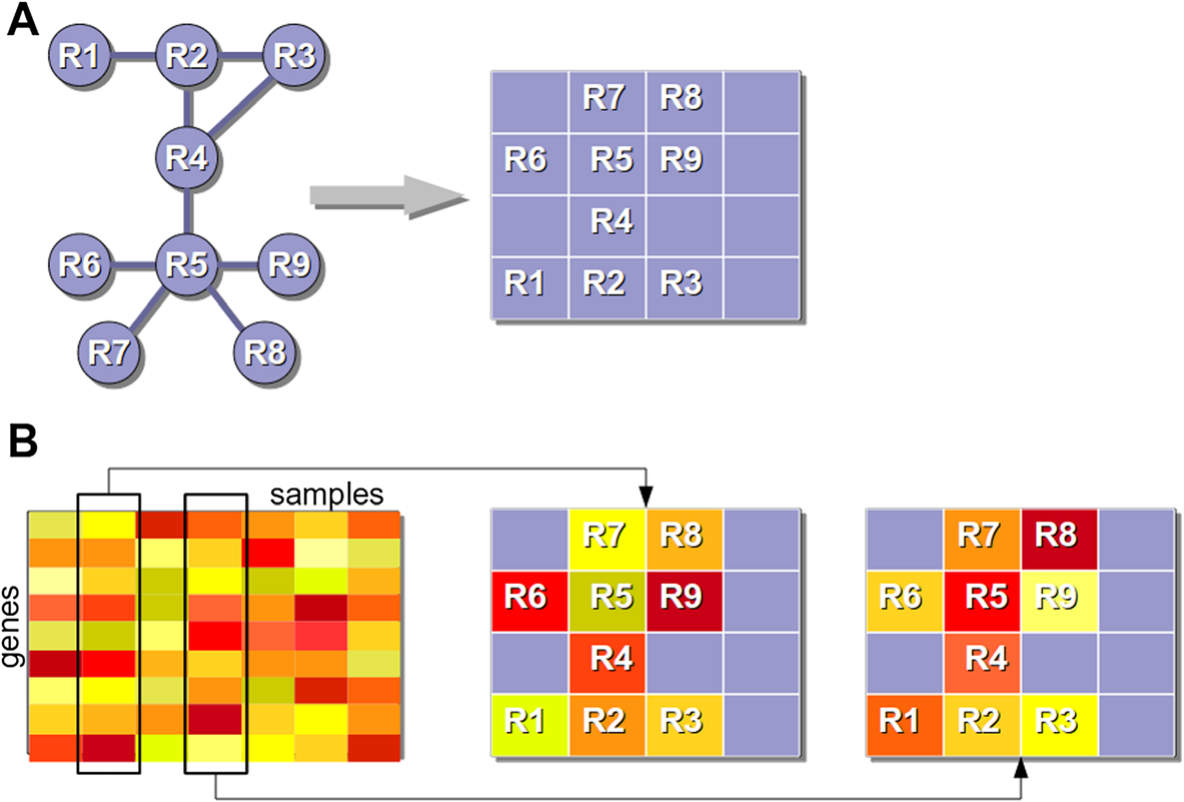
**Mapping of pathways and expression data.** Schematic representations of **(A)** the embedding of pathways into compact 2D lattice grids and **(B)** gene expression data mapped onto to the embedded pathways. In this toy example, R1 to R9 represent metabolic reactions.

The workflow consists of two major steps: the preprocessing of pathway information and the analysis of expression data. We briefly summarize the two steps (for more details, see Schramm *et al*. [1]):

Step 1 (preprocessing of pathway information): To run a PathWave analysis which involves the use of wavelet transforms (see below), compact 2D grid representations of pathways are required. Curated pathway maps are translated into networks in which nodes represent metabolic reactions and edges link two reactions if one reaction produces a metabolite that serves as a substrate for the other. Edges in signaling pathways are derived from direct interactions of the corresponding signaling proteins. These networks are described by their binary adjacency matrices {*A*_*ij*_}_*i*,*j* ∈ *V*_ where *V* is the set of nodes and *A*_*ij*_ = 1 if nodes *i* and *j* are joined by an edge. The sparse adjacency matrices (*A*_*ij*_ = 0 for most pairs of nodes) are optimally rearranged and embedded into smaller 2D, regular square lattice grid representations, best preserving the neighborhood characteristics of the reactions by placing adjacent nodes (*A*_*ij*_ = 1) of the original network as close to each other as possible on the lattice grid (see Figure 1A). For this, the overall Manhattan distance on the lattice grid between neighboring nodes of the network is minimized using integer linear programming techniques. These optimal 2D grid representations need to be computed only once for a given set of pathways and can be saved for later use. We calculated and integrated preprocessed pathway sets for several species into the new PathWave R package (see below), such that this step can be skipped for these organisms.

Step 2 (analysis of expression data): To identify pathways that are differentially regulated between two conditions or subsets of gene expression data, PathWave first associates gene expression levels with metabolic reactions or signaling proteins, thus mapping the expression data of each individual sample onto the 2D lattice grids of the pathways (see Figure 1B). Then, Haar wavelet transforms are applied to each optimized grid to explore every possible expression pattern of neighboring reactions or signaling proteins within an embedded pathway and to identify statistically significant discriminative patterns between samples of the two different conditions [1]. We used Haar wavelet transforms to systematically apply low pass and high pass filters from fine grain to coarse resolutions [11].

**Figure 2:**
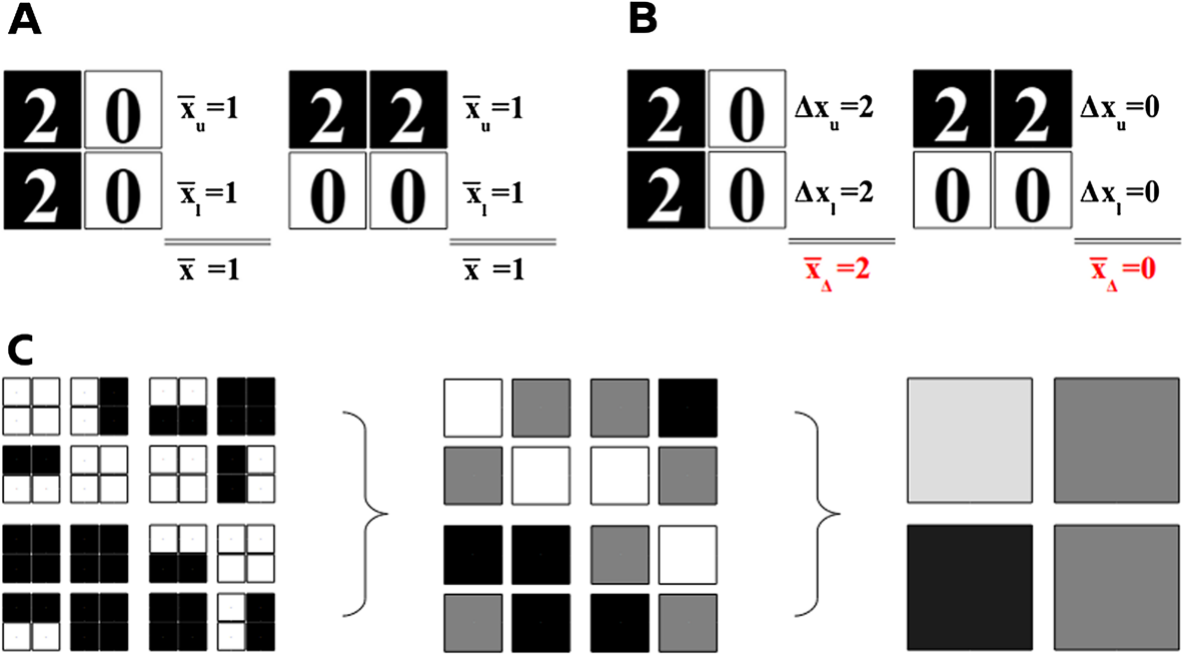
**Schematic representation of the Haar wavelet transformation. (A, B)** Representation of a local (high frequency) change in an expression pattern that is **(A)** not detected by low pass filters (here, 1st feature; mean of means) but **(B)** detected by high pass filters (here, 3rd feature; mean of differences). **(C)** Step-wise reduction of the resolution by averaging (1st feature) of 2 × 2 sections.

Namely, a 2D expression grid—corresponding to the expression data of a single sample mapped onto a particular pathway (as exemplified in Figure 1B)—is divided into disjoint sections of 2 × 2 pixels (i.e. nodes), e.g. a 2D grid of size 8 × 8 is divided into 16 disjoint sections. Grids with odd sizes (e.g. 3 × 3, 4 × 5, 5 × 5) are extended with rows and/or columns of zeros to allow their division into disjoint 2 × 2 sections. For each section, all combinations of row-wise and column-wise mean and differences, respectively, are calculated, yielding 4 combined quantitative features for each 2 × 2 section: 1st) mean of the mean of the upper and mean of the lower row; 2nd) difference of the mean of the upper and the mean of the lower row; 3rd) mean of the difference of the upper and the difference of the lower row; and 4th) difference of the difference of the upper and the difference of the lower row (see Figure 2A and B for an example). When these features are determined for all sections of the grid, all mean of means features (1st) are taken to construct a new, smaller averaged grid which is again grouped into 2 × 2 sections to be transformed into features in the same manner, again yielding 4 quantitative features for every section. This procedure is repeated until no further averaging is possible (see Figure 2C), thus computing quantitative low and high pass features from fine grain (initial 2D grid) to coarse resolutions (averaged grids after repeated grouping). For more details on this Haar wavelet transformation, please see Mallat [11]. For each pathway, the features at all resolutions, obtained from the wavelet transformation, are used to identify features that can significantly discriminate between samples of the two conditions or subsets.

Thus, Haar wavelets suit to detect both globally dysregulated pathways (low-pass filter) and switch-like behaviors within the pathways, which significantly affect only small subnetworks (high-pass filter). To overcome the rigidity of wavelet transforms, PathWave covers any possible combination of neighboring nodes by implementing a one-step frameshift concept that applies the transform to the original grids and to grids shifted by one row, one column, or by both. Finally, pathways are ranked according to their most discriminative expression pattern, being it at a fine (e.g. metabolic switch in a sub-network) or coarse grain resolution (e.g. global up- or down-regulation of an entire pathway). This ranking is done by statistical significance which is obtained by repeated random sampling of the samples (i.e., patients; drawing without replacement) [1]. Namely, for each pathway a Gumbel distribution (extreme value distribution) is fitted to the pathway-scores obtained for the randomly shuffled samples. *P*-values of the pathways are determined from the respective fitted curves and the actual scores obtained for unpermuted samples, and are finally corrected for multiple testing. The robustness of both the estimated *P*-values of single pathways and the resulting overall pathway ranking increase with the number of random samplings. An assessment of their stability demonstrates a good to excellent robustness already at a rather modest number of 1,000 to 10,000 random samplings (see Additional file 1).

It is to note that an expression pattern composed of multiple genes (regarding an entire pathway or a sub-network) may significantly discriminate between two conditions (e.g. tumor and normal tissue) even when the single genes do not appear to be differentially expressed after multiple testing correction (e.g. using *t*-tests). Due to the identification of such potentially relevant patterns within pathways, PathWave can be more sensitive than common gene set enrichment approaches.

### Improved embedding of metabolic pathways in 2D lattice grids

The current PathWave version uses a new, up to 10^3^ times faster branch-and-cut algorithm to embed metabolic and signaling pathways into compact 2D lattice grids, on which wavelet transforms can be applied, and to allow for optimal arrangements of larger instances [12]. The new method to optimally arrange every KEGG/BiGG pathway *G* = (*V*,*E*) (represented by an undirected, unweighted network or graph *G* with nodes *V* and edges *E*) into a compact 2D lattice grid computes an embedding of *G* into the lattice grid with the minimum possible total Manhattan distance (edge length) of nodes (metabolic reactions or signaling proteins) that are adjacent in *G*. The method is based on a novel integer programming formulation that models the pairwise distances between the nodes on the grid, instead of their absolute positions used in the previous version [1]. Clearly, not every distance combination is a feasible solution: We say that an integral distance vector {*d*_*uv*_}_*uv* ∈ *E*_ is embeddable, if there exists a 2D arrangement of the nodes *V* on the grid such that the Manhattan distance of *u* and *v* is exactly *d*_*uv*_ for every edge *uv*.

For every unordered pair {*u*,*v*} of adjacent nodes *u* and *v*, we set the distance variable *x*_*uvk*_ to 1 if *k* is less than or equal to the Manhattan distance of *u* and *v*. We set it to 0 otherwise. Since for fixed *u* and *v* the number of variables being 1 is exactly the Manhattan distance of *u* and *v*, the latter can be written as the sum of all variables *x*_*uvk*_ over *k*. To obtain the total length of a pathway's embedding (sum of all Manhattan distances) one has to additionally sum over all edges *uv* that constitute the pathway. This is done in the objective function (1) of the following integer programming formulation whose goal is to minimize the pathway's total length in the embedding:

(1) $$\min\sum_{\mathrm{uv}\in E}\sum_{k=1}^{d_{\text{max}}}x_{\mathrm{uvk}}$$

subject to

(2) $$\sum_{\mathrm{uv}\in E_{H}}\left(x_{\mathrm{uv}d_{\mathrm{uv}}}-x_{\mathrm{uv}\left(d_{\mathrm{uv}}+1\right)}\right)\leq \left|E_{H}\right|-1$$

for all sub graphs *H* = (*V*_*H*_,*E*_*H*_) of *G* and all distance vectors *d* on *H* that are not embeddable, and to

(3) $$x_{\mathrm{uvk}}\geq x_{\mathrm{uv}\left(k+1\right)}$$

for all *uv* ∈ *E*, 1 ≤ *k* ≤ *d*_*max*_ - 1, where all *x* variables are required to be binary.

The objective function (1) minimizes the total edge length of the embedding. The constraints in equation (2) ensure that the optimal distance vector is embeddable. They are the reason for the choice of the binary distance variables *x*, as these constraints cannot be modeled with integer variables directly taking the distance values (i.e., *d*_*uv*_). Since their number is exponential, we do not add them to the model from the beginning, but separate them during the branch-and-cut algorithm. The constraints in equation (3) ensure consistency of the *x* variables: if the Manhattan distance is at least *k* + 1, then it must be also at least *k*. The maximum possible distance *d*_*max*_ can be set to the diagonal diameter of the lattice grid. As *d*_*max*_ is usually significantly lower in practice, it is estimated by our algorithm via an initial series of tests. Moreover, the branch-and-cut algorithm features a simple primal construction heuristic. It produces approximate solutions even for large instances.

### Compatibility and programming languages

PathWave 2.1 is compliant with the latest KEGG KGML version (v0.7.1) and an additional Perl script can be used to translate Systems Biology Markup Language (SBML) files from the BiGG database [7,8] to compatible KGML-like files. While the parsing of the KGML pathway files to produce adjacency matrices is implemented in R, the integral linear program (LP) that optimizes grid arrangements is written in C++, using CPLEX (ILOG, Gentilly, France) as an LP solver.

### Improved user-interface

We made the PathWave user interface more flexible and easier to use. Now, running a PathWave analysis (step 2) requires a single function call in R, as shown in the following simplified example:

pwres < - pathWave.run(preprocessed.tag = "KEGG.hsa", input.sampleclasses = "sample_classes.tsv", input.exprdata = "expr_data.tsv", param.numperm = 10000, param.pvalue.correction.method = "Bonferroni", param.pvalue.threshold = 0.001, param.kegg.only_metabolism = TRUE)

Expression data and the definition of the two sample classes, or conditions, can either be specified as file names or passed as R data.frame objects and/or factor objects. The preprocessed pathway information (from step 1) to be used is specified as well as the number of permutations to be performed for statistical testing. When using KEGG, the search can optionally be limited to metabolic pathways. Additionally, we provide a simple function to build URLs for querying the KEGG web server at http://www.genome.jp/kegg/ to obtain graphical pathway maps with color-coded metabolic reactions or signaling proteins according to their up- or down-regulation:

urls < - pathWave.getColorKEGGMapURLs(pwres$results.filtered, preprocessed.tag = "KEGG.hsa")

For convenience, the R package of the new version includes seven sets of preprocessed pathways for six species: KEGG pathways for *H. sapiens*, *M. musculus*, *D. melanogaster*, *D. rerio*, *C. elegans*, and *E. coli* and pathways extracted from the human metabolic model from BiGG [7,8].

## Results and discussion

### First case study: analyzing regulation of metabolic pathways in lung cancer

Lung adenocarcinoma is the most common type of lung cancer, and lung cancer is the world-wide leading cause of cancer-related deaths in males [13]. Here, we have used PathWave and analyzed a gene expression dataset derived from a large-scale RNA sequencing study [14] to identify metabolic pathways showing significant dysregulation between lung adencarcinomas and patient matched normal controls of tumor adjacent tissues. As expected, metabolism of lung cancer cells showed profound differences to normal cell metabolism (Table 1). One of the characteristic changes in metabolism of tumor cells is an increased uptake and utilization of glucose and fermentation into lactate [15], already described by Otto Warburg in the 1920s [16]. Accordingly, PathWave identified glycolysis to be significantly dysregulated (Bonferroni corrected *P* < 1E-16), along with the citrate cycle (TCA cycle; *P* < 1E-16). Notably, in contrast to most reactions of the two pathways, some key reactions were down-regulated as shown in Additional file 1: Figure S1 and Additional file 1: Figure S2: *FBP1* and *FBP2* encode fructose-1,6-bisphosphatase 1 and 2 (EC 3.1.3.11) and showed significantly lower expression in tumor cells than in adjacent normal tissue (*P* = 2.5E-7 and *P* = 5.8E-3, respectively). The reaction catabolized by these enzymes converts D-fructose 1,6-bisphosphate to D-fructose 6-phosphate and opposes the metabolic flux during glycolysis (gluconeogenesis). In turn, the inverse reaction catabolized by phosphofructokinase (EC 2.7.1.11) was upregulated to allow high glycolytic flux. Phosphoenolpyruvate carboxykinase (PCK1, EC 4.1.1.32) is the main control point for extracting metabolites from the TCA cycle for gluconeogenesis, and this enzyme was downregulated while lactate production was upregulated.

**Table 1:** Metabolic pathways with significant dysregulation in lung cancer

**Pathway**	***P*** *	Up*	No_ch*	Down*
Glycolysis/Gluconeogenesis	< 1E-16	20	9	4
Citrate cycle (TCA cycle)	< 1E-16	12	9	2
Fructose and mannose metabolism	< 1E-16	10	8	1
Fatty acid metabolism	< 1E-16	15	11	8
Steroid biosynthesis	< 1E-16	7	11	11
Ubiquinone and other terpenoid-quinone biosynthesis	< 1E-16	8	0	3
Purine metabolism	< 1E-16	63	22	6
Pyrimidine metabolism	< 1E-16	55	16	5
Alanine, aspartate and glutamate metabolism	< 1E-16	16	9	2
Cysteine and methionine metabolism	< 1E-16	11	12	2
Arginine and proline metabolism	< 1E-16	16	26	6
Tyrosine metabolism	< 1E-16	5	20	19
Tryptophan metabolism	< 1E-16	9	18	10
N-Glycan biosynthesis	< 1E-16	19	9	5
Amino sugar and nucleotide sugar metabolism	< 1E-16	15	20	2
Inositol phosphate metabolism	< 1E-16	5	14	11
Glycosylphosphatidylinositol(GPI)-anchor biosynthesis	< 1E-16	8	5	2
Glycerophospholipid metabolism	< 1E-16	13	17	11
Arachidonic acid metabolism	< 1E-16	13	8	15
Sphingolipid metabolism	< 1E-16	7	17	3
Glycosphingolipid biosynthesis - lacto and neolacto series	< 1E-16	31	13	3
Glycosphingolipid biosynthesis - ganglio series	< 1E-16	4	8	11
Pyruvate metabolism	< 1E-16	9	14	4
One carbon pool by folate	< 1E-16	16	8	2
Vitamin B6 metabolism	< 1E-16	7	0	4
Nicotinate and nicotinamide metabolism	< 1E-16	5	8	5
Folate biosynthesis	< 1E-16	9	8	3
Porphyrin and chlorophyll metabolism	< 1E-16	9	7	1
Drug metabolism - cytochrome P450	< 1E-16	8	15	23
Drug metabolism - other enzymes	< 1E-16	15	7	6
Metabolic pathways	< 1E-16	418	388	201
Primary bile acid biosynthesis	8.77E-15	12	18	16
Butanoate metabolism	1.75E-14	8	4	2
Valine, leucine and isoleucine degradation	2.63E-14	11	24	2
Glutathione metabolism	6.14E-14	13	9	0
Starch and sucrose metabolism	2.10E-13	7	14	6
Steroid hormone biosynthesis	3.86E-13	32	45	21
Glycine, serine and threonine metabolism	2.05E-12	12	15	2
Lysine degradation	7.22E-12	10	6	0
Fatty acid elongation in mitochondria	9.09E-11	12	6	7

*Up is the number of up-regulated reactions in lung adenocarcinoma when compared to normal controls, down the number of down-regulated reactions; and no_ch the number of reactions without notable changes. P is the Bonferroni corrected *P*-value for the pathway pattern.

We detected a less known but nonetheless interesting metabolic switch in lung adenocarcinomas, namely the increased conversion of tryptophan to kynurenine by tryptophan 2,3-dioxygenase (EC 1.13.11.11) and indoleamine 2,3-dioxygenase (EC 1.13.11.52), both of which were upregulated (see Additional file 1: Figure S3). Kynurenine has been shown to induce immunosuppression and aid tumor cells to evade immune surveillance in tumors [17-19]. Interestingly, we also found a significant pattern in glycosylphosphatidylinositol (GPI) anchor biosynthesis. Biosynthesis of GPI anchors is essential for appropriately locating GPI-anchored proteins onto cellular membranes. GPI transamidase (GPI-T) is in the downstream part of the pathway representation by KEGG. The upregulation of GPI-T has been described in tumorigenesis and its genes suggested as biomarkers [20]. Interestingly, the pattern we found showed particularly the upper part of GPI biosynthesis to be upregulated (significant upregulation of *PIG*-*C*: *P* = 1.46E-11 (Bonferroni corrected), *DPM2*: *P* = 7.04E-11, *PIG*-*L*: P = 0.005, *PIG*-*W*: *P* = 5.21E-8 and *PIG*-*M*: *P* = 1.02E-13 encoding enyzmes processing UDP-N-acetylglucosamine into GPI anchor (GlcN)1 (Ino(acyl)-P)1 (Man)1).

For comparison, we performed the same analysis using pathways extracted from the human metabolic model of the BiGG database (see Additional file 1: Table S1). The obtained results were generally in line with those obtained for KEGG and differences were mostly due to pathways that were differently represented in these databases.

To demonstrate that PathWave's extension of pathway analysis to the identification of significantly dysregulated subnetworks is more sensitive than common gene set enrichment approaches, we applied GSEA [9] and DAVID [10] to the same dataset for metabolic KEGG pathways. Using DAVID, only two pathways were significant after correction for multiple testing (Bonferroni), namely aminoacyl-tRNA biosynthesis (*P* = 6.46E-4) and purine metabolism (*P* = 9.80E-3), and only six pathways had a nominal (uncorrected) *P*-value of less than 0.05 (see Additional file 1: Table S2). For GSEA, three pathways were identified at nominal *P* < 0.05 but none was significant after correction for multiple testing (see Additional file 1: Table S3).

### Second case study: pathways associated with aging in *Drosophila melanogaster*

As a second case study, we analyzed microarray expression data of *D. melanogaster* with an extended lifespan and controls [21] to identify pathways being relevant for aging. We compared gene expression profiles of 8 samples from naturally long-living specimen with 8 samples from normal specimen at optimal nutrition. For the *Drosophila* study signaling and metabolic pathways were considered. The two most prominent pathways obtained (see Table 2) are indeed associated with aging in other species:

**Table 2:** Pathways with significant dysregulation in long-lived *D. melanogaster*

**Pathway**	***P*** *	Up*	No_ch*	Down*
Starch and sucrose metabolism	2.71E-03	6	11	1
Circadian rhythm - fly	7.90E-03	3	5	0
Glycerophospholipid metabolism	1.02E-02	9	25	3
Tyrosine metabolism	1.44E-02	4	5	0
Nicotinate and nicotinamide metabolism	2.23E-02	3	6	0
Glycine, serine and threonine metabolism	2.97E-02	4	14	3
Jak-STAT signaling pathway	3.15E-02	1	11	2
Ubiquinone and other terpenoid-quinone biosynthesis	3.26E-02	7	4	0
Endocytosis	4.52E-02	6	16	1
Purine metabolism	4.87E-02	23	49	9

*Up is the number of up-regulated reactions in long-lived flies when compared to normal controls, down the number of down-regulated reactions; and no_ch the number of reactions without notable changes. P is the Bonferroni corrected *P*-value for the pathway pattern.

(i) Starch and sucrose metabolism (Additional file 1: Figure S4) is central for starch digestion which has been reported to increase with increasing age in chicken [22,23], suggesting that it is more effectively metabolized. In long lived specimen of *Drosophila* this could in principle be driven by an increased expression of genes encoding for enzymes digesting starch into glucose (we identified upregulation of EC 2.4.1.1, EC 3.2.1.33, see Additional file 1: Figure S4). (ii) We detected a significant pattern in the pathway for circadian rhythm. The disruption of the circadian clock has been associated with aging and morbidity [24]. Notably, expression of the *clock* gene was higher in *D. melanogaster* with an extended lifespan, consistent with the recent observation that the disruption of the encoded protein (dCLK in Additional file 1: Figure S5) reduces lifespan in mice [25].

## Methods

### KEGG pathways

KEGG pathways [2] were extracted from the corresponding KGML files obtained from the KEGG website (download: April 14, 2011) for the following species: *H. sapiens* (human), *M. musculus* (mouse), *D. melanogaster* (fly), *D. rerio* (zebrafish), *C. elegans* (worm), and *E. coli*. The KGML files were preprocessed to build pathway networks in which nodes represent metabolic reactions or signaling proteins (depending on the type of pathway) that are linked by an edge if one reaction produces a metabolite that serves as a substrate for another or one signaling protein interacts with another, respectively. For each of these pathway networks an optimally arranged 2D grid representation was produced as described in Results.

### Extraction of pathway maps from the human metabolic model

The compartmentalized version of the Human recon 1 metabolic model [7] was obtained from the BiGG database [8]. The SBML file was parsed to extract subsystems (e.g. “Alanine and Aspartate Metabolism”) that were interpreted as pathways and converted to KGML-like XML files. These files were preprocessed in the same way as the pathways obtained from KEGG (see above).

To focus on functionally relevant interactions, several exceedingly common metabolites (like H_2_O) were ignored. Roughly, metabolites were excluded if they were involved in more than eight reactions. The final list was manually curated to (i) retain some specific, informative metabolites even if they were involved in more than eight reactions; and to (ii) exclude additional uninformative metabolites even if they were involved in eight or less reactions. Additional file 1: Table S4 lists all metabolites that were excluded from the model and thus from the extracted subsystems/pathways.

### RNA-seq data for lung cancer

Gene expression levels for 77 lung adenocarcinomas and patient matched normal tissue were obtained from a recently published large-scale RNA sequencing study [14] (GEO accession: GSE40419). Expression levels were given in reads per kilobase of exon model per million mapped reads (RPKM) [26]. We mapped the HUGO gene symbols obtained from GEO to their corresponding Entrez/NCBI gene IDs [27] (mapping downloaded on August 31st, 2013) and restricted our analysis to genes with unique mappings (20,817 genes). For these, the lowest non-zero RPKM value of the dataset was added to all expression levels as a pseudocount, such that expression levels could be log2-transformed for use with PathWave.

### Gene expression data for the *Drosophila* study

For this case study, we used a very recent expression data set for *D. melanogaster*[21] (Gene Expression Omnibus identifier: GSE36582). The dataset consisted of 46 samples of adult females of normal lifespan (22 samples) and adult females of a long-lived, natural variant of *D. melanogaster* (24 samples; obtained by selection for starvation resistant specimens). Samples were selected at two time points (middle age sampled at 90% survival; and old age sampled at 10% survival) and for three adult diets (malnutrition, optimal food, and overfeeding). For more details, see Doroszuk *et al*. [21]. For our analysis, we selected 16 samples of animals with optimal nutrition: 8 samples for normal specimen (four at each time point) and 8 samples for long-lived specimen (four at each time point). We used the data as normalized in [21]. We mapped probeset IDs of the Affymetrix Drosophila Genome 2.0 Array to gene IDs using the producers annotation information (na31). Probesets that mapped to more than one EntrezGene ID were discarded. Expression profiles from multiple probesets mapping to the same gene ID were averaged yielding one single expression profile for each of the 12,578 genes analyzed.

### Mapping of gene expression data to metabolic reactions

PathWave applies Haar wavelet transforms to compact 2D grid representations of metabolic pathways. The represented nodes are metabolic reactions, not genes. Hence the gene expression values were associated with these reactions. This is done by averaging the expression values of all genes coding for enzymes that are required for catabolizing the reaction. The resulting reaction expression profiles are z-transformed over the sample set and mapped onto the compact 2D grid representations of each pathway.

### PathWave analyses

For the *Drosophila* case study, we performed the PathWave analysis on both metabolic and signaling KEGG pathways using default parameters with the exception of performing 10,000 (default: 1,000) random shufflings of phenotype labels (long-lived *D. melanogaster* and normal controls) for statistical testing. Final pathway *P*-values were corrected for multiple testing using the Bonferroni correction. For the lung cancer case study, we performed the PathWave analysis on metabolic pathways from KEGG and the human metabolic model from BiGG. Having more samples and thus more statistical power, we additionally required at least 10 genes/reactions to be up- or downregulated for a pathway to be selected and lowered the threshold of the Bonferroni corrected *P*-value from 0.05 to 0.001.

### Comparison with DAVID and GSEA

To compare the sensitivity of PathWave with other, common approaches we used the RPKM expression values of 77 lung adenocarcinomas and their matched normal tissue, which was the larger dataset of our case studies.

We identified 4,078 significantly upregulated and 3,065 significantly downregulated genes (Wilcoxon signed-rank test; Bonferroni-corrected *P*-value < 0.05) which were tested for significant pathway enrichment using DAVID (http://david.abcc.ncifcrf.gov/). The background was the set of all 20,817 genes we had used for the PathWave analysis. The functional enrichment analysis was performed with default parameters. Results were extracted only for metabolic KEGG pathways and *P*-value correction for multiple testing was applied accordingly. For GSEA (http://www.broadinstitute.org/gsea/), we used only annotations from metabolic KEGG pathways. Statistical significance was evaluated by comparison with 10,000 random shufflings of phenotype labels (tumor or normal) of the 154 samples.

## Conclusions

Many patho-physiological clinical courses come along with major changes in cell physiology affecting single pathways or even largely remodelling signaling and the entire cellular metabolism, such as in the case of cancers [15,28]. Even the dysregulation of only a few genes in a pathway can lead to a substantial regulatory switch that alters the output of a signaling process or redirects the metabolic flux in the cell. This may be related to aging or lead to growth advantages driving tumorigenesis, particularly when dysregulated genes are connected in the same subnetwork of a pathway and their functionality is closely linked. A good example found in this study, is the conversion of starch to glucose for which we observed a regulatory switch enabling improved digestion of starch in long-lived *Drosophila* specimen. Unfortunately, most pathway analysis methods require a considerably high number of genes to be dysregulated within a pathway in order to suggest the pathway as functionally relevant for the disease. These approaches do not consider significant regulatory patterns interlinked at smaller scales. For the purpose of identifying not only globally dysregulated pathways but in particular important switches in pathways, we have developed PathWave. Here, we presented a considerably improved version that, among other changes, uses a novel LP formulation to optimally embed pathway networks into compact 2D lattice grids and provides a more flexible and user-friendly interface including ready-to-use pre-arranged 2D grid representations of pathway collections for now six organisms.

We used PathWave to identify pathways that play central roles in human lung cancer and longevity of *D. melanogaster*. A comparison to commonly used pathway and gene set analysis methods showed PathWave to be considerably more sensitive. In contrast to common approaches, it regards pathways also when they contain only small numbers of significantly dysregulated genes, which may potentially constitute functionally relevant pathway switches, suggesting follow up investigations in the wet-lab.

## Availability and requirements

**Project name:** PathWave

**Project homepage:**http://www.ichip.de/software/pathwave.html

**Operating system(s):** Platform independent

**Programming language:** R, C++, Perl

**Other requirements:** For running PathWave on expression data: R version 2.14 or higher, standard CRAN R packages (XML, e1071, gtools, evd), standard Bioconductor R packages (multtest, RCurl, genefilter); for embedding pathways into compact 2D lattice grids: CPLEX, GridArranger v1.0 (available from the project homepage), ABACUS v2.4-alpha (available from the project homepage).

**License:** GNU GPL

**Any restrictions to use by non**-**academics:** none

## Competing interests

The authors declare that they have no competing interests.

## Authors’ contributions

RK, RMP and RE designed the study; SW, MO and GR designed the new grid optimization method; SW and NR implemented the new grid optimization method; RMP and GS implemented the new PathWave R package; RMP performed the data analysis; RMP, RK and SW wrote the manuscript. All authors read and approved the final manuscript.

## Supplementary Material

Additional file 1 Supplementary text, figures and tables. Click here for file
