# Supplementary material for: Network topology-based detection of differential gene regulation and regulatory switches in cell metabolism and signaling
Source: BMC Syst Biol. 2014 May 16;8:56. doi: 10.1186/1752-0509-8-56 (PMC4031158; doi:10.1186/1752-0509-8-56)
Supplement: Additional file 1 — Supplementary text, figures and tables. [file 1752-0509-8-56-S1.pdf]

Network topology-based detection of differential gene regulation and regulatory switches in cell metabolism and signaling

Rosario M Piro, Stefan Wiesberg, Gunnar Schramm, Nico Rebel, Marcus Oswald, Roland Eils, Gerhard Reinelt, Rainer König

Supplementary information

Content

Table S1: Pathway analysis with PathWave using the human pathways from BiGG (page 3)

Table S2: Pathway analysis with DAVID for the lung cancer dataset on metabolic KEGG pathways (page 4)

Table S3: Pathway analysis with GSEA for the lung cancer dataset on metabolic KEGG pathways (page 4)

Table S4: Metabolites removed from the human metabolic model from BiGG (page 4)

Figure S1: Glycolysis in lung adenocarinoma compared to normal tissue (page 6)

Figure S2: TCA cycle in lung adenocarinoma compared to normal tissue (page 7)

Figure S3: Tryptohpan metabolism in lung tumors compared to paired adjacent normal tissue (page 8)

Figure S4: Starch and sucrose metabolism in long-lived flies compared to flies with normal lifespan (page 9)

Figure S5: Circadian rhythm in long-lived D. melanogaster compared to D. melanogaster with normal lifespan (page 10)

Stability of P-values and robustness of pathway rankings (page 11)

Figure S6: Stability of pathway *P*-values as measured by their variation coefficients (page 12)

Figure S7: Recall of pathway significance as a function of the mean *P*-value (page 12)

Figure S8: Robustness of pathway rankings (page 13)

Table S1: Pathway analysis with PathWave using the human pathways from BiGG

Human metabolic model subsystem (pathway)	P*	up*	no_ch*	down*
Arginine_and_Proline_Metabolism	< 1E-16	6	5	5
Blood_Group_Biosynthesis	< 1E-16	23	12	10
Cholesterol_Metabolism	< 1E-16	11	11	9
Glycerophospholipid_Metabolism	< 1E-16	5	13	9
Glycine,_Serine,_and_Threonine_Metabolism	< 1E-16	10	1	1
Glycolysis_Gluconeogenesis	< 1E-16	11	3	3
Glycosylphosphatidylinositol__GPI__anchor_biosynthesis	< 1E-16	15	19	2
IMP_Biosynthesis	< 1E-16	10	0	0
Inositol_Phosphate_Metabolism	< 1E-16	4	18	9
Keratan_sulfate_biosynthesis	< 1E-16	38	18	3
N_Glycan_Biosynthesis	< 1E-16	25	6	17
Nucleotides	< 1E-16	39	41	5
Pyruvate_Metabolism	< 1E-16	5	10	7
Sphingolipid_Metabolism	< 1E-16	22	16	30
Steroid_Metabolism	< 1E-16	16	16	7
Transport,_Extracellular	< 1E-16	49	38	26
Transport,_Mitochondrial	< 1E-16	16	2	1
Tryptophan_metabolism	< 1E-16	6	15	11
Tyrosine_metabolism	< 1E-16	3	24	19
Eicosanoid_Metabolism	3.82E-14	2	2	11
Chondroitin_____heparan_sulfate_biosynthesis	4.77E-14	3	19	19
Bile_Acid_Biosynthesis	1.15E-13	16	10	11
Folate_Metabolism	1.62E-13	9	21	1
Carnitine_shuttle	2.96E-13	31	31	31
Pentose_Phosphate_Pathway	2.85E-12	5	5	5
Chondroitin_sulfate_degradation	4.02E-12	10	22	1
Keratan_sulfate_degradation	5.16E-09	26	45	0
Fatty_acid_oxidation	7.23E-04	0	2	25

*up is the number of metabolic reactions up-regulated in lung adenocarcinoma; down the number of down-regulated reactions; and no_ch the number of reactions without notable changes. P is the Bonferroni corrected P-value for the entire pathway.

Table S2: Pathway analysis with DAVID for the lung cancer dataset on metabolic KEGG pathways

Pathway name	Fold enrichment	P (nominal)*	P (Bonferroni)
Aminoacyl-tRNA biosynthesis	1.95	7.26E-06	6.46E-04
Purine metabolism	1.40	1.10E-04	9.80E-03
N-Glycan biosynthesis	1.57	4.54E-03	4.04E-01
Phenylalanine metabolism	1.88	4.80E-03	4.27E-01
Tyrosine metabolism	1.53	1.09E-02	9.70E-01
Pyrimidine metabolism	1.28	3.46E-02	1.00E+00

*Only pathways with a nominal (uncorrected) P-value < 0.05 are shown.

Table S3: Pathway analysis with GSEA for the lung cancer dataset on metabolic KEGG pathways

Pathway	NES	P (nominal)*	P (Bonferroni)
Pyrimidine metabolism	1.69	4.45E-03	3.96E-01
Aminoacyl-tRNA biosynthesis	1.59	2.03E-02	1.00E+00
Purine metabolism	1.36	4.10E-02	1.00E+00

*Only pathways with a nominal (uncorrected) P-value < 0.05 are shown.

Table S4: Metabolites removed from the human metabolic model from BiGG

Compartment: Cytosol (_c)				
M_h_c	M_h2o_c	M_atp_c	M_pi_c	M_adp_c
M_na1_c	M_coa_c	M_o2_c	M_nadp_c	M_nadph_c
M_ppi_c	M_nad_c	M_nadh_c	M_amp_c	M_co2_c
M_nh4_c	M_crn_c	M_h2o2_c	M_gln_DASH_L_c	M_hco3_c
M_dcdp_c	M_accoa_c	M_ser_DASH_L_c	M_ala_DASH_L_c	M_ump_c
M_udp_c	M_gly_c	M_gdp_c	M_cys_DASH_L_c	M_dadp_c
M_dgdp_c	M_dudp_c	M_glc_DASH_D_c	M_dtdp_c	M_cmp_c
M_cl_c	M_amet_c	M_R2coa_hs_c	M_ahcys_c	M_thr_DASH_L_c
M_pyr_c	M_paps_c	M_ctp_c	M_pap_c	M_malcoa_c
M_gdpmann_c	M_asn_DASH_L_c	M_so4_c	M_Rtotalcoa_c	M_mthgxl_c
M_gthrd_c	M_for_c	M_datp_c	M_dag_hs_c	M_cdp_c
M_ac_c	M_thf_c	M_pmtcoa_c	M_pchol_hs_c	M_dgtp_c
M_chol_c	M_ACP_c	M_utp_c	M_udpg_c	M_uacgam_c
M_gtp_c	M_f6p_c	M_dctp_c	M_asp_DASH_L_c	M_adn_c

M_uri_c	M_k_c	M_dttp_c	M_dhap_c	M_dcmp_c
M_arachd_c	M_retn_c	M_retinol_c	M_pro_DASH_L_c	M_pail_hs_c
M_oh1_c	M_mal_DASH_L_c	M_lys_DASH_L_c	M_lald_DASH_D_c	M_g3p_c
M_fum_c	M_cytd_c	M_acald_c		
Compartment: EndoplasmicReticulum (_r)				
M_h_r	M_h2o_r	M_nadp_r	M_nadph_r	M_o2_r
M_dolp_U_r	M_dolp_L_r	M_nad_r	M_nadh_r	M_udp_r
M_pe_hs_r	M_dolmanp_U_r	M_dolmanp_L_r	M_udpglcur_r	M_dag_hs_r
M_pi_r	M_glc_DASH_D_r	M_chsterol_r		
Compartment: Extraorganism (_e)				
M_na1_e	M_h_e	M_h2o_e	M_hco3_e	M_gln_DASH_L_e
M_cl_e	M_ser_DASH_L_e	M_cys_DASH_L_e	M_pi_e	M_glc_DASH_D_e
M_ala_DASH_L_e	M_thr_DASH_L_e	M_gly_e	M_glu_DASH_L_e	M_asn_DASH_L_e
M_k_e				
Compartment: GolgiApparatus (_g)				
M_h_g	M_udp_g	M_udpgal_g	M_uacgam_g	M_paps_g
M_pap_g	M_cmp_g	M_cmpacna_g	M_h2o_g	M_gdp_g
M_gdpfuc_g	M_man_g	M_udpacgal_g	M_udpglcur_g	
Compartment: Lysosome (_l)				
M_h2o_l	M_h_l	M_so4_l	M_acgam_l	M_glu_DASH_L_l
M_pi_l	M_glcur_l	M_acgal_l	M_gal_l	
Compartment: Mitochondria (_m)				
M_h_m	M_h2o_m	M_coa_m	M_nad_m	M_nadh_m
M_atp_m	M_fad_m	M_fadh2_m	M_adp_m	M_nadp_m
M_nadph_m	M_crn_m	M_pi_m	M_o2_m	M_co2_m
M_dudp_m	M_dadp_m	M_dgdp_m	M_dcdp_m	M_dtdp_m
M_occoa_m	M_pyr_m	M_ppi_m	M_ppcoa_m	M_dutp_m
M_datp_m	M_amp_m	M_gtp_m	M_gdp_m	M_hco3_m
Compartment: Nucleus (_n)				
M_h_n	M_atp_n	M_adp_n	M_h2o_n	M_udp_n
M_pi_n	M_dcdp_n	M_cdp_n	M_dcmp_n	
Compartment: Peroxisome (_x)				
M_h2o_x	M_coa_x	M_o2_x	M_h_x	M_h2o2_x
M_accoa_x	M_nadh_x	M_nad_x		

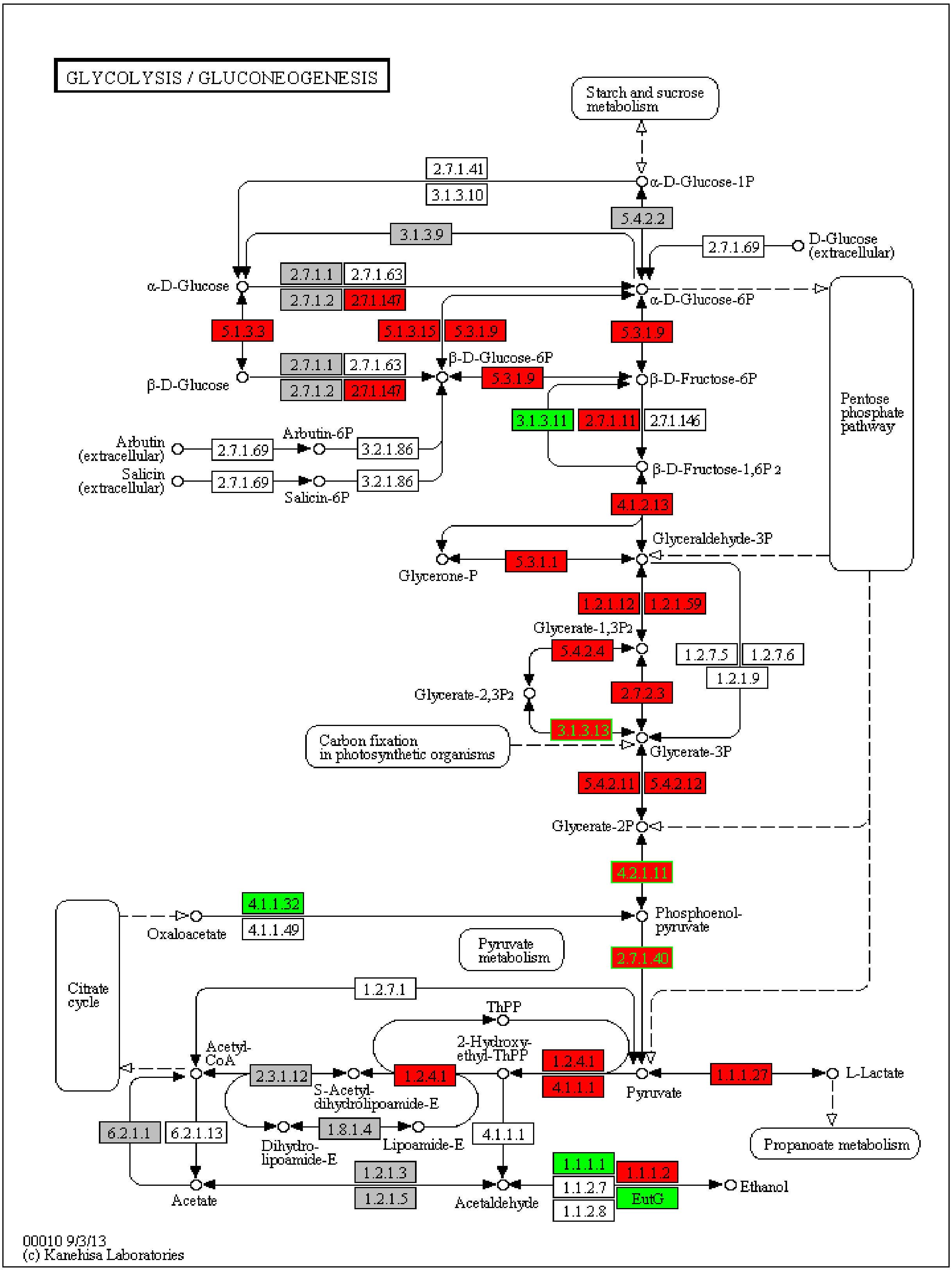

Figure S1: Glycolysis in lung adenocarinoma compared to normal tissue

red = up-regulated; green = down-regulated; grey = no notable change
(template is taken from http://www.genome.jp/kegg/)

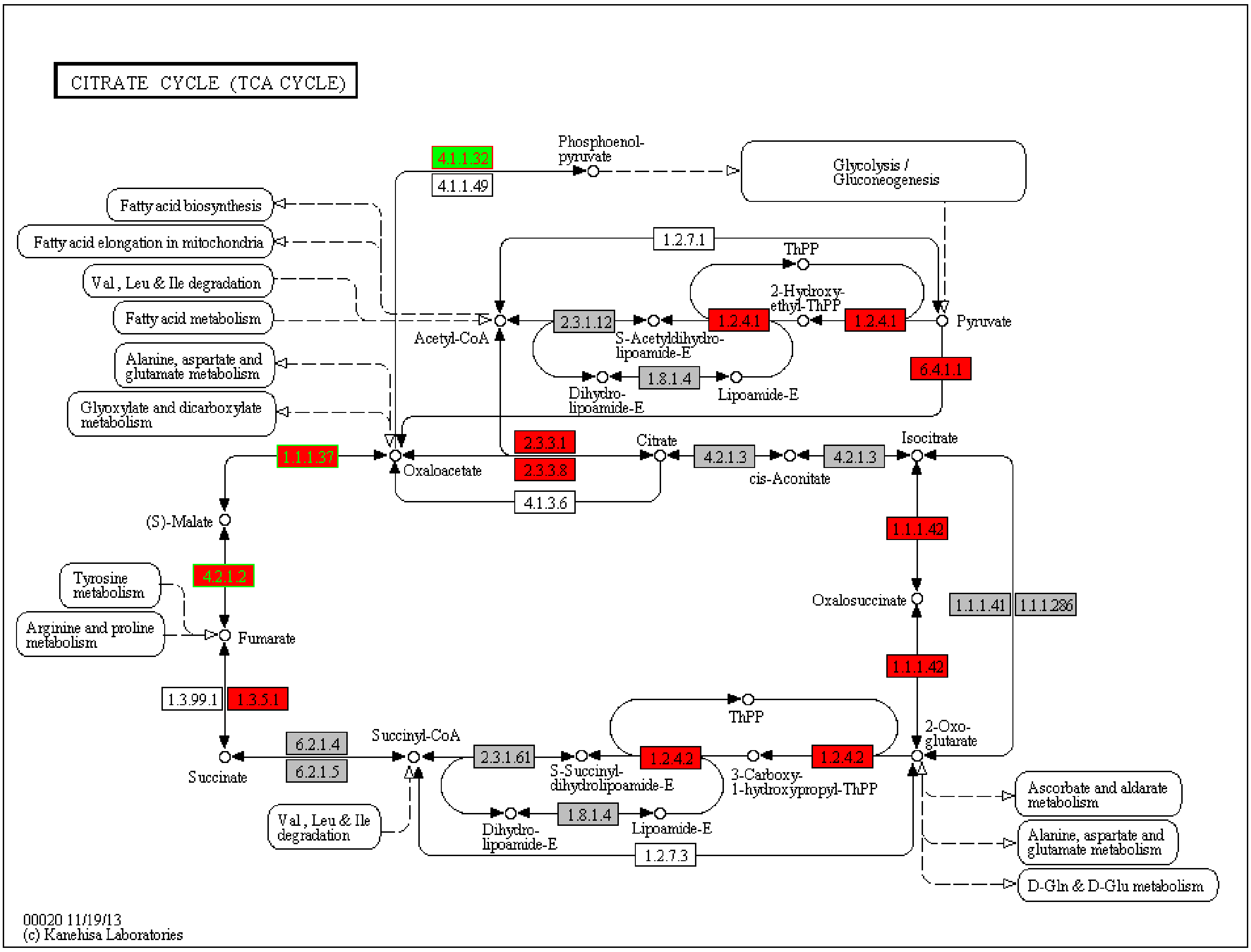

Figure S2: TCA cycle in lung adenocarinoma compared to normal tissue

red = up-regulated; green = down-regulated; grey = no notable change.
(template is taken from http://www.genome.jp/kegg/)

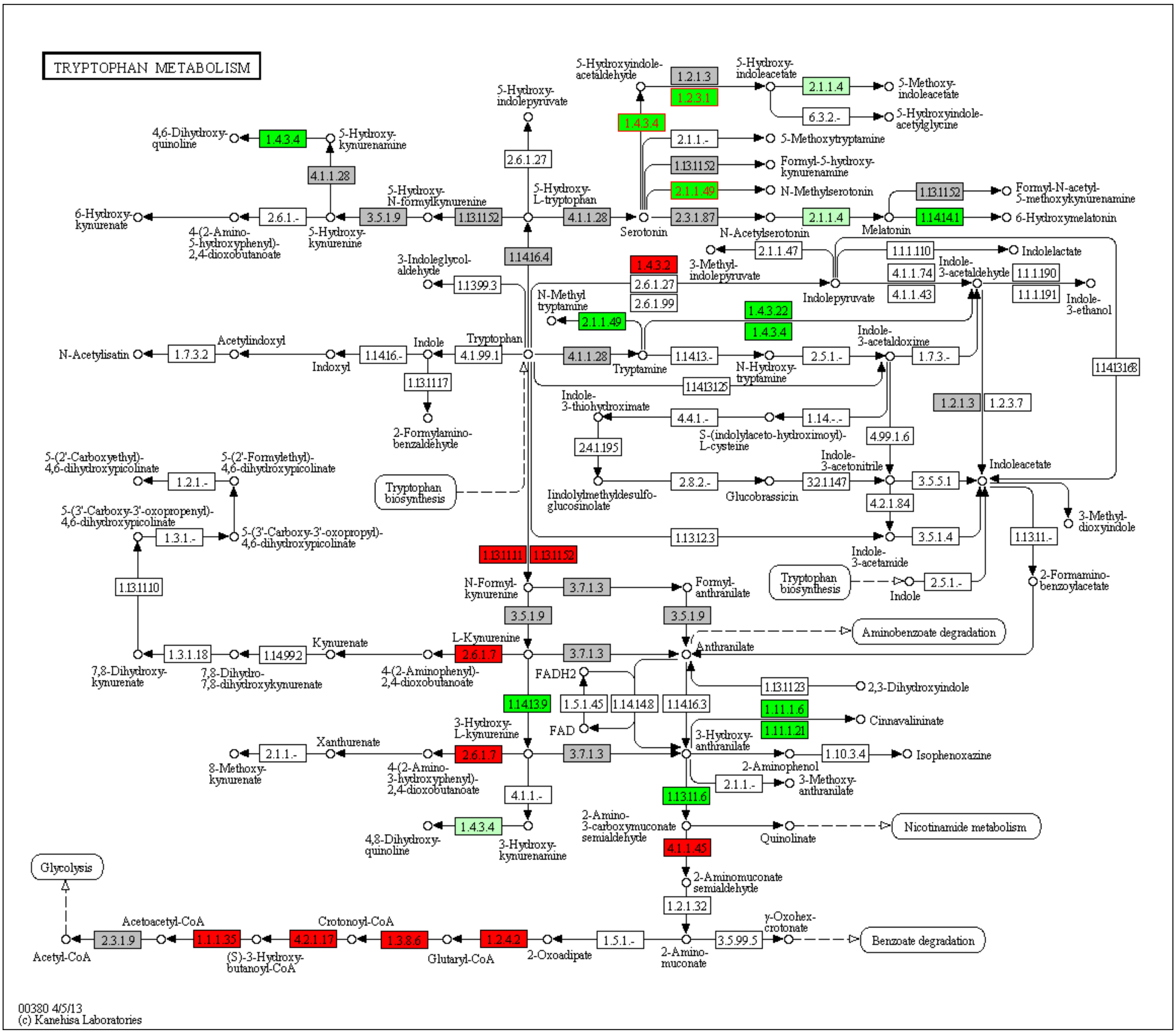

Figure S3: Tryptohpan metabolism in lung tumors compared to paired adjacent normal tissue

red = up-regulated; green = down-regulated; gray = no notable change.
(template is taken from http://www.genome.jp/kegg/)

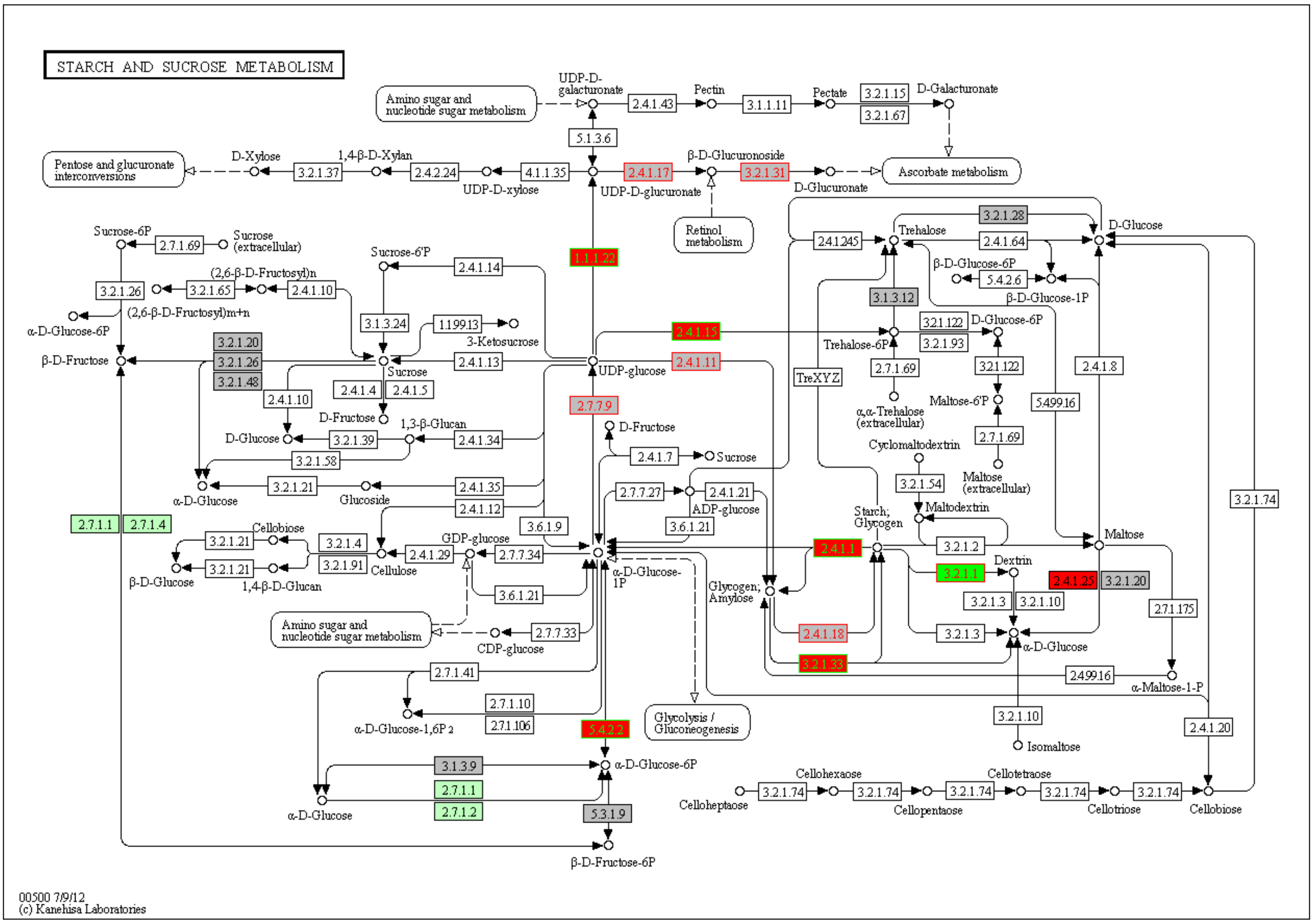

Figure S4: Starch and sucrose metabolism in long-lived flies compared to flies with normal lifespan

red = up-regulated in long lived flies; green = down-regulated; grey = no notable change; light green = enzyme present in flies, but no expression data available (template is taken from http://www.genome.jp/kegg/)

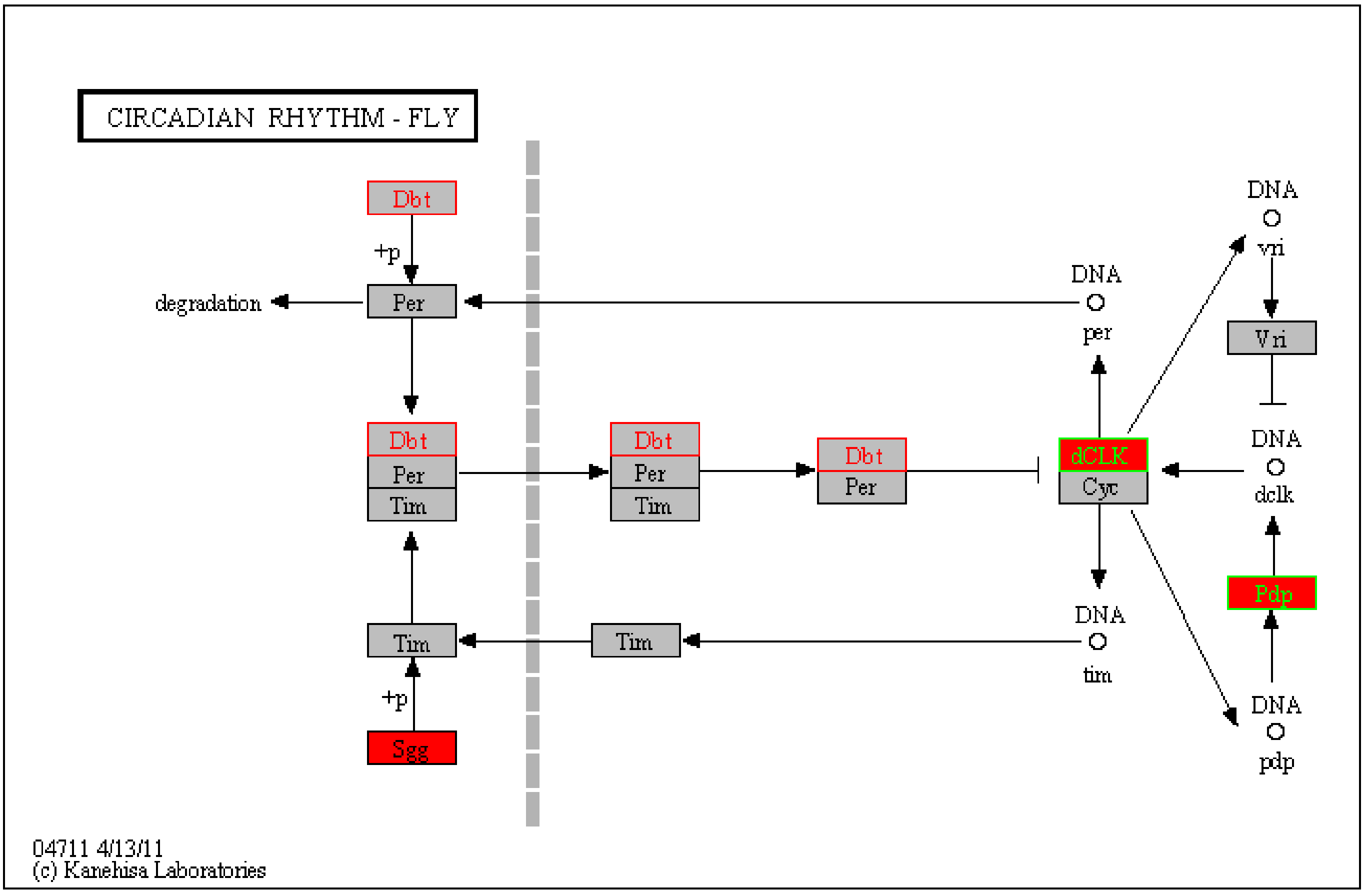

Figure S5: Circadian rhythm in long-lived *D. melanogaster* compared to *D. melanogaster* with normal lifespan

red = up-regulated; grey = no notable change (template is taken from http://www.genome.jp/kegg/)

Stability of *P*-values and robustness of pathway rankings

The pathway *P*-values estimated by PathWave suffer from a problem which is generally known for Monte Carlo resampling methods: the empirically determined *P*-values depend on what random permutations are actually executed and may thus change from execution to execution, even with the same dataset, because the executed number of random permutations (which gives an approximate *P*-value estimate) is usually much lower than the total number of possible permutations (which would give an accurate *P*-value estimate).

We therefore evaluated the stability and reproducibility of the pathway *P*-values and the resulting pathway rankings performing 100 identical PathWave runs for case study 2 (D. melanogaster aging; see article) and using three measures:

1. The variation coefficients (or coefficients of variation) of *P*-values of each pathway, defined as the ratio σ/μ of the standard deviation σ to the mean μ. Multiplication of this ratio by 100 measures the standard deviation as a percentage of the mean and indicates how strongly *P*-values variate over 100 identical runs.

2. The "recall" of pathways (i.e. the number of times a pathway is declared significant) as a function of its mean *P*-value, indicating how frequently pathways with a *P*-value close to the threshold are possibly missed or wrongly declared significant.

3. The average pairwise Spearman correlation between the 100 pathway rankings obtained from the 100 identical PathWave runs, indicating how stable pathway rankings are across multiple identical runs.

To evaluate how these measures depend on the number of Monte Carlo samplings (parameter `numperm`) used for *P*-value estimation, we executed PathWave 100 times (identical runs) for each of `numperm`=100, `numperm`=1000, and `numperm`=10000.

The results show that for 10,000 random samplings (used in the article), the *P*-values of single pathways (Figure S6) are highly stable (standard deviation < 5% of mean for most pathways), as are the overall pathway rankings (Figure S8; median pairwise Spearman correlation of 0.9996) over 100 runs. Moreover, the results indicate an excellent recall (Figure S7; right). As expected, pathways with an average *P*-value very close to 0.05 (here ca. ± 2%) are declared significant in roughly 50% of the runs. Pathways with average *P*<0.045 have a recall of >95% (100% for average *P*<0.04).

For 1,000 random samplings (used as a default in the PathWave R package), the results indicate a slightly lower but still sufficient stability. The *P*-values of most pathways have standard deviations of <20% of their mean (Figure S6), and the obtained pathway rankings are nearly as stable as for 10,000 random samplings (Figure S8; median pairwise Spearman correlation of 0.9970). Although pathways with an average *P*-value of close to 0.05 are recalled with slightly more difficulty (Figure S7; middle), their overall recall is still good (>95% for average *P*<0.04; 100% for average *P*<0.03).

For 100 random samplings, in contrast, both the *P*-values (Figures S6 and S7) and the pathway rankings (Figure S8) are not sufficiently stable.

Overall, these anlyses suggest that 1,000 random samplings (default in the R package) are sufficient and offer a reasonably high confidence in the obtained results. The 10,000 random samplings used in the article allow for an even higher confidence as well as an excellent stability.

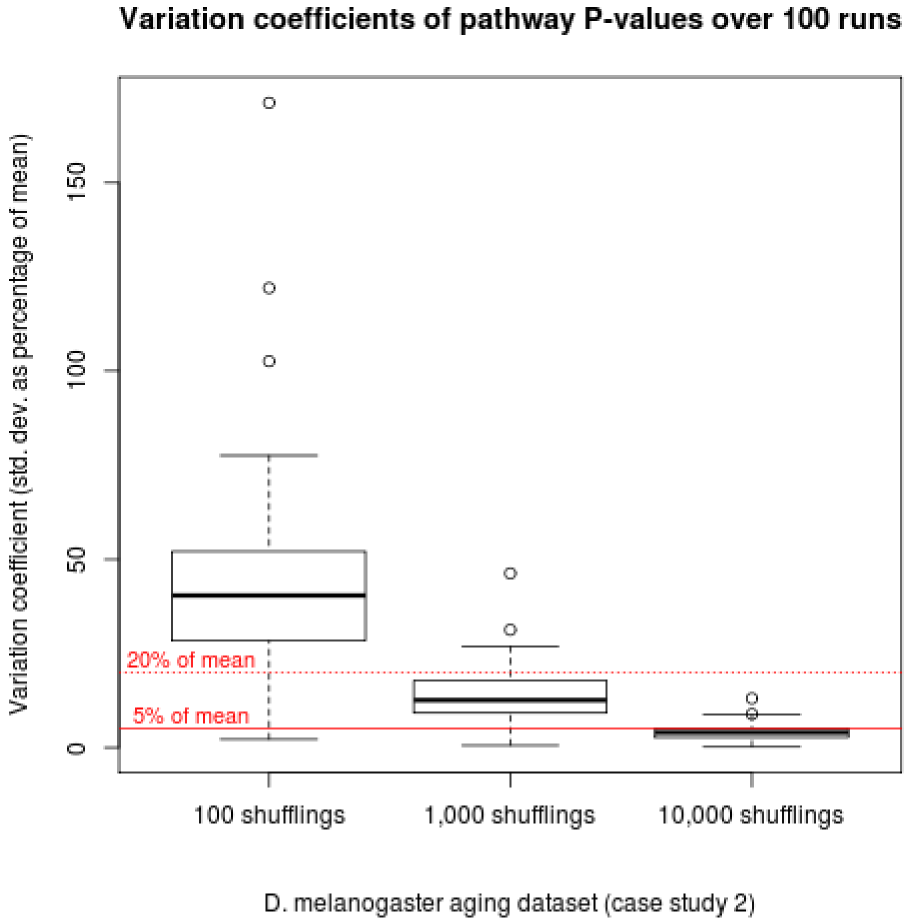

Figure S6: Stability of pathway *P*-values as measured by their variation coefficients
The variation coefficients were determined for each single pathway over 100 identical PathWave runs. Boxplots depict the distribution of variation coefficients of the pathways for 100 (left), 1000 (middle), and 10,000 random samplings (`numperm`).

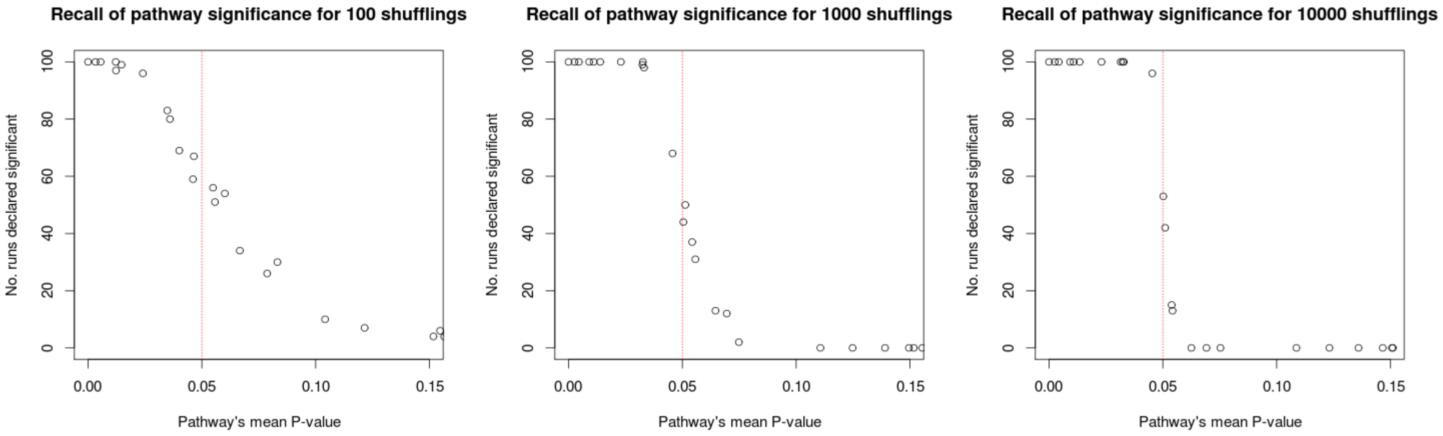

Figure S7: Recall of pathway significance as a function of the mean *P*-value
The number of times a pathway is declared significant (at a *P*-value threshold of 0.05) in 100 identical PathWave runs is drawn as a function of the pathway's mean *P*-value for 100 (left), 1000 (middle), and 10,000 (right) random samplings (`numperm`).

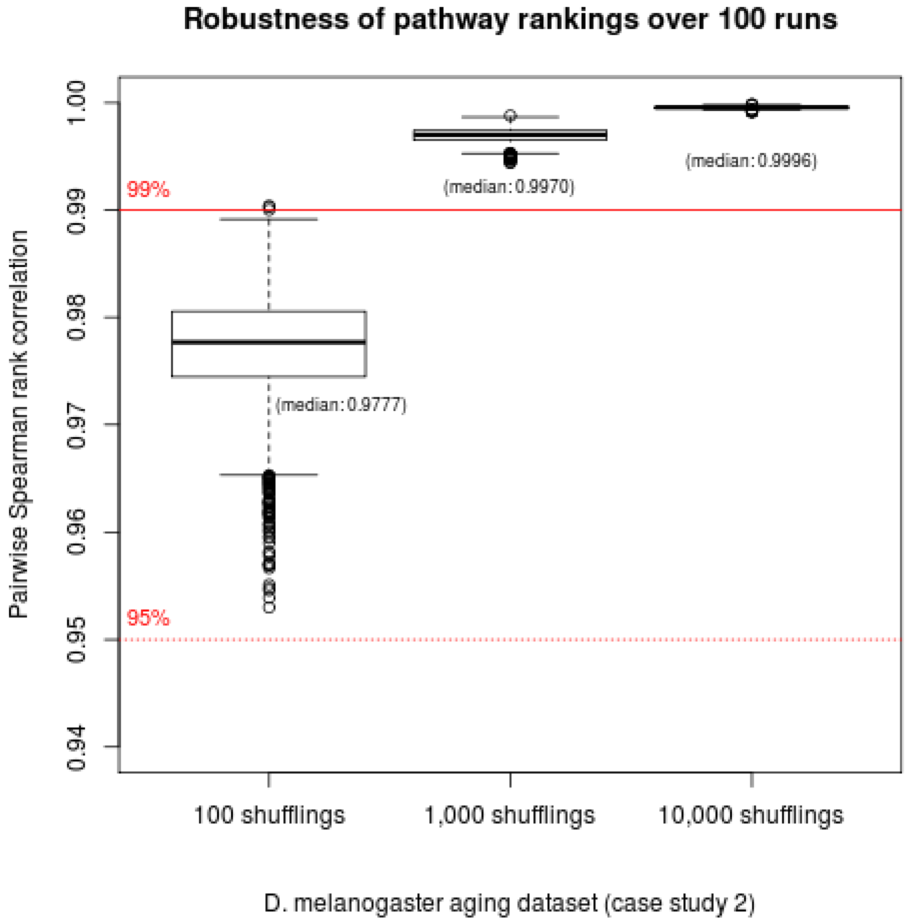

Figure S8: Robustness of pathway rankings

The robustness of pathway rankings over 100 identical PathWave runs is shown as boxplots of the rankings' pairwise Spearman rank correlations for 100 (left), 1000 (middle) and 10,000 random samplings (`numperm`).
